# Association between internalized stigma and depression among HIV-positive persons entering into care in Southern India

**DOI:** 10.7189/jogh.07.020403

**Published:** 2017-12

**Authors:** Brian T Chan, Amrose Pradeep, Lakshmi Prasad, Vinothini Murugesan, Ezhilarasi Chandrasekaran, Nagalingeswaran Kumarasamy, Kenneth H Mayer, Alexander C Tsai

**Affiliations:** 1 Division of Infectious Diseases, Brigham and Women’s Hospital, Boston, Massachusetts, USA; 2 Harvard Medical School, Boston, Massachusetts, USA; 3 Y. R. Gaitonde Centre for AIDS Research and Education, Chennai, India; 4 Division of Infectious Diseases, Beth Israel Deaconess Medical Center, Boston, Massachusetts, USA; 5 Fenway Health, Boston, Massachusetts, USA; 6 MGH Global Health, Massachusetts General Hospital, Boston, Massachusetts, USA; 7 Chester M. Pierce, MD Division of Global Psychiatry, Massachusetts General Hospital, Boston, Massachusetts, USA

## Abstract

**Background:**

In India, which has the third largest HIV epidemic in the world, depression and HIV–related stigma may contribute to high rates of poor HIV–related outcomes such as loss to care and lack of virologic suppression.

**Methods:**

We analyzed data from a large HIV treatment center in southern India to estimate the burden of depressive symptoms and internalized stigma among Indian people living with HIV (PLHIV) entering into HIV care and to test the hypothesis that probable depression was associated with internalized stigma. We fitted modified Poisson regression models, adjusted for sociodemographic variables, with probable depression (PHQ–9 score ≥10 or recent suicidal thoughts) as the outcome variable and the Internalized AIDS–Related Stigma Scale (IARSS) score as the explanatory variable.

**Findings:**

521 persons (304 men and 217 women) entering into HIV care between January 2015 and May 2016 were included in the analyses. The prevalence of probable depression was 10% and the mean IARSS score was 2.4 (out of 6), with 82% of participants endorsing at least one item on the IARSS. There was a nearly two times higher risk of probable depression for every additional point on the IARSS score (Adjusted Risk Ratio: 1.83; 95% confidence interval, 1.56–2.14).

**Conclusions:**

Depression and internalized stigma are highly correlated among PLHIV entering into HIV care in southern India and may provide targets for policymakers seeking to improve HIV–related outcomes in India.

To help end the worldwide AIDS epidemic by 2030, the Joint United Nations Programme on HIV/AIDS proposed a set of Fast Track or “90–90–90” targets to be achieved by 2020: the diagnosis of 90% of all people living with HIV (PLHIV), the provision of antiretroviral therapy (ART) to 90% of those diagnosed, and the achievement of an undetectable viral load for 90% of those on treatment [1]. India, which has the third largest HIV epidemic in the world with 2.1 million PLHIV [2], has dramatically scaled–up access to ART over the last decade [3,4]. However, high rates of loss to HIV care suggest that India is far from achieving the 90–90–90 targets. In one cohort study in Andhra Pradesh, only 31% of patients diagnosed with HIV ultimately achieved virologic suppression [5]. Similarly, the overall drop–out rate at a large HIV care center in Tamil Nadu was 38 per 100 person–years [6]. Finally, in a nationwide cohort of men who have sex with men (MSM) and people who inject drugs (PID), only 10% of HIV–infected cohort participants were on ART and virologically suppressed [7]. These estimates suggest that India is far from achieving the goal to eliminate AIDS.

Little is known about the reasons for loss to HIV care in India after diagnosis. One study of loss to HIV care focused on clinical predictors such as CD4^+^ cell count and weight [8]. More recently, a study of Indian MSM and PID found that certain clinic–based factors, failure to disclose one’s serostatus to others, and depressive symptoms were associated with decreased odds of linkage to HIV care [9]. Beyond this study, little is known about how psychosocial factors such as depression and HIV–related stigma affect loss to HIV care in India.

In other settings, particularly in sub–Saharan Africa, HIV–related stigma has been associated with psychological distress and depression [10,11] and poorer ART adherence among PLHIV [12,13]. Similarly, depression among PLHIV has been associated with increased transmission risk [14,15], greater CD4^+^ count declines [16,17], reduced ART adherence [18], and more rapid progression to AIDS and death [17,19,20]. Importantly, depression is a modifiable risk factor, as treatment of depression can result in reduced risk of HIV transmission [21] and improved ART adherence and virologic suppression [22]. Some studies have suggested that depression treatment should be combined with behavioral interventions to maximize improvements in HIV–related outcomes [23-25].

Compared to other low– and middle–income countries (LMICs) in which stigma and depression have been studied more extensively (eg, countries in sub–Saharan Africa), India features a markedly different socio–cultural environment and an epidemic that is highly concentrated among female sex workers, MSM, and PID [2]. As such, one cannot assume that findings on stigma and depression from other LMICs are applicable to the Indian context. Both HIV–related stigma [26–28] and depression [29–31] have been found to be highly prevalent among Indian PLHIV. Although some studies have shown an association between stigma and depression among Indian PLHIV [26,32,33], these studies included PLHIV both in and out of care at ART centers. If stigma and depression inhibit PLHIV from seeking care or contribute to loss to care after enrollment, then estimates based on mixed samples could potentially overstate the association between stigma and depression. Understanding the association between stigma and depression specifically among PLHIV entering into HIV care is particularly important, as this step in the HIV care continuum represents an opportunity to initiate interventions to attenuate their potential negative effects on HIV–related outcomes. If stigma and depression are closely correlated among PLHIV entering into care and initiating ART, then multi–faceted behavioral interventions targeting both depression as well as drivers of stigma may be needed to keep these PLHIV in care and virologically suppressed.

To help address these gaps in knowledge, we conducted a study of adults entering into care at a large HIV treatment center in Chennai, India. We hypothesized that *internalized stigma* (the internalization and acceptance of negative attitudes towards PLHIV and subsequent development of self–defacing beliefs [34]) and *depression* would be highly prevalent in this population and that internalized stigma and depression were highly correlated. The aims of this study were to 1) estimate the burden of internalized stigma and depression and 2) estimate the association between internalized stigma and depression.

## METHODS

### Study setting and procedures

This cross–sectional study was conducted at the Y.R. Gaitonde Centre for AIDS Research and Education (YRG CARE) in Chennai, India. YRG CARE is one of the largest private organizations providing HIV care in India, having treated over 20 000 PLHIV since its founding in 1993. Chennai is the capital of Tamil Nadu state and one of the epicenters of the HIV epidemic in India [35]. As in other areas of India, the HIV epidemic in Tamil Nadu is concentrated, with an overall prevalence of 0.31% but a higher prevalence among female sex workers, MSM, and PID (between 2.4% and 9.5%) [2,36,37]. The majority of patients at YRG CARE come from Tamil Nadu and Andhra Pradesh states. Most patients at YRG CARE self–identify as heterosexual [29], as there are other organizations in Chennai which are perceived as specializing in care for sexual and gender minorities.

Patients were eligible for participation in the study if they were at least 18 years of age, HIV–infected, and newly entering into HIV care at YRG CARE between January 2015 and May 2016. Interviews were about 20 minutes long and were conducted by one of two female HIV counselors in the patient’s preferred language (Tamil, Telugu, or English). All study materials were written in English, professionally translated into Tamil or Telugu, and back–translated to English to ensure fidelity to the original. Study staff obtained signed, or in case of illiteracy, thumbprint–marked informed consent documents from all participants. No remuneration was given. Potential participants were informed both verbally and through informed consent documents that refusal to participate would not impact the care that they would receive. Ethical approval for study procedures was obtained from the Institutional Review Boards of Fenway Health (Boston, Massachusetts, USA) and YRG CARE.

### Measures

We administered questionnaires to PLHIV at their first clinical visit to YRG CARE after HIV diagnosis. To assess for depressive symptoms, we included the Patient Health Questionnaire–9 (PHQ–9) [38,39], which has been previously translated into Tamil and Telugu and validated in India [40,41]. Higher PHQ–9 scores (range, 0–27) signify greater levels of depression symptom severity. Study participants were considered to screen positive for probable depression if they either 1) returned a PHQ–9 score of greater than or equal to 10 or 2) endorsed the item “thoughts that [one] would be better off dead or of hurting [one]self in some way”, as recommended by Kroenke and Spitzer [42].

To measure internalized stigma, we included the six–item Internalized AIDS–Related Stigma Scale (IARSS) [43]. We chose to focus on internalized stigma given a growing consensus that internalized stigma is an important predictor of HIV–related outcomes [44]. The IARSS is the most commonly used measure of internalized stigma in the literature, has been validated in multiple settings [45,46], and is relatively brief compared to other commonly–used stigma scales such as the Berger HIV Stigma Scale [47] and the HIV/AIDS Stigma Instrument – PLWA [48]. Two items relate to concerns about disclosure and four items relate to feelings of shame and/or self–hatred (Table 1). Responses were elicited on a binary scale (yes/no); scale scores represent the sum total of endorsed items (range 0–6). Like most HIV–related stigma scales, given that the degree of stigma is measured along a continuum, there is no clear prevalence cutoff for determining who is or is not “stigmatized”. Although this was the first known use of the IARSS in India, questions with similar wording have been validated in India [28,32]. Further, we pilot–tested the questions on five Tamil and five Telugu speakers to ensure comprehensibility and face and content validity.

**Table 1:** Characteristics of patients by gender

Characteristic	Overall (n = 521)	Women (n = 217)	Men (n = 304)	t–test / χ^2^ statistic	*P*
**Sociodemographic and clinical variables**					
Age, mean (SD), y	39.6 (8.8)	37.1 (7.6)	41.3 (9.1)	5.50	<0.001*
Achieved more than primary education, %	63.7	56.7	68.8	7.98	0.005*
Married, %	67.0	53.5	76.6	30.8	<0.001*
Employed, %	75.6	45.2	97.4	187.2	<0.001*
Urban residence, %	35.3	33.6	36.5	0.46	0.50
Tamil speaker, %	19.6	17.1	21.4	1.51	0.22
Telugu speaker, %	76.4	81.6	72.7	5.52	0.02*
Heterosexual, %	96.4	98.6	94.7	5.43	0.02*
Baseline CD4^+^ cell count, mean (SD)	347.7 (493.3)	396.8 (345.7)	314.0 (571.1)	1.64	0.10
**Substance use variables**					
Current alcohol use, %	14.0	0	24.0	60.6	<0.001*
Injection drug use, %	0.4	0	0.7	1.43	0.23
**Depressive symptoms and internalized stigma**					
PHQ–9 score, mean (SD)	2.8 (3.3)	2.8 (3.2)	2.7 (3.4)	0.41	0.68
Probable depression, %	9.6	10.6	8.9	0.43	0.51
IARSS score, mean (SD)	2.4 (1.7)	2.3 (1.6)	2.5 (1.7)	1.46	0.15
It is difficult to tell people about my HIV infection, %	80.6	80.2	80.9	0.04	0.83
Being HIV positive makes me feel dirty, %	28.6	26.3	30.3	0.99	0.32
I feel guilty that I am HIV positive, %	26.3	24.4	27.6	0.67	0.41
I am ashamed that I am HIV positive, %	13.4	10.6	15.5	2.57	0.11
I sometimes feel worthless because I am HIV positive, %	15.6	12.0	18.1	3.60	0.06
I hide my HIV status from others, %	77.7	76.0	79.0	0.62	0.43

y – years, SD – standard deviation, PHQ–9 – Patient Health Questionnaire–9, IARSS – Internalized AIDS–Related Stigma Scale

*P < 0.05.

Sociodemographic variables of interest, collected as part of the standard YRG CARE patient questionnaire and clinical protocol, included gender, age, educational attainment, marital status (married vs other), employment status, rural/urban residence, language (Tamil/Telugu/other), sexual orientation (“homosexual,” “heterosexual,” or “bisexual”), alcohol use (“yes” vs “no” or “stopped”), and injection drug use (IDU) (“present use or previous use” vs “never use”).

### Statistical analysis

We used descriptive statistics to characterize the sample and to estimate the prevalence of probable depression, frequency of responses to the individual internalized stigma items, and mean PHQ–9 and IARSS scores. Responses were compared by gender using chi–square tests for categorical variables and t–tests for continuous variables. We then used Pearson correlation coefficient to estimate the correlation between internalized stigma (IARSS) and depressive symptoms (PHQ–9). We also dichotomized IARSS at the median value (0–2 vs 3–6) and compared probable depression by high and low internalized stigma score using a chi–square test.

Next, we used Poisson regression models [49,50], modified with robust estimates of variance [51,52], to estimate the association between probable depression and internalized stigma (IARSS score). Following Zou [49], the incidence rate ratios were interpreted as risk ratios. We estimated both unadjusted and adjusted models, with the latter including sociodemographic variables as potential confounders. A statistically significant regression coefficient for IARSS was considered evidence that an association existed between internalized stigma and probable depression. As an alternative parameterization, we fitted a multivariable linear regression model with depressive symptoms (PHQ–9 score) as the outcome of interest.

To explore the possibility of bias from unobserved confounders of the relationship between internalized stigma and depressive symptoms, we used the sensitivity analysis detailed in Oster [53]. This procedure assumes a value for the maximum R–squared from a regression model and calculates a value for the relative degree of confounding by unobserved vs observed variables (the “delta”) that would result in a regression coefficient equal to zero. We selected a maximum R–squared value of 1.3 multiplied by the R–squared obtained in the multivariable linear regression model, as this is the level of robustness consistent with findings from randomized controlled trials [53].

## RESULTS

524 persons entering into HIV care between January 2015 and May 2016 were enrolled into the study, including 304 men, 217 women, and 3 Hijra (transgender women). Because of the low number of Hijra enrolled, we dropped these three observations from the analyses. No one refused entry into the study, although not all eligible patients may have been approached because of variable interviewer availability. Participant characteristics are stratified by gender in **Table 1**. 502 (96%) self–reported as heterosexual. The mean age was 40 years (standard deviation (SD) 9 years). Prevalence of self–reported IDU was low (<1%). Telugu was the primary language of 76% of participants, with a majority of the remaining participants reporting Tamil as their primary language. The mean baseline CD4^+^ cell count (available for 394 participants) was 348 cells/mm^3^ (SD, 493).

The prevalence of probable depression was 10% and the mean PHQ–9 score was 2.8 (SD, 3.3). The scale reliability coefficient for the PHQ–9 was 0.80. The mean IARSS score was 2.4 (SD, 1.7). 427 (82%) participants endorsed at least one of the six IARSS items and 33 (6%) of participants endorsed all six IARSS items. The two IARSS items most commonly endorsed were related to concerns about disclosure: “I hide my HIV status from others” (78%) and “It is difficult to tell people about my HIV infection” (81%). The scale reliability coefficient for the IARSS was 0.79. The Pearson correlation coefficient between IARSS and the PHQ–9 was 0.47, indicating a correlation of moderate magnitude. 23% (44/192) of participants with an IARSS score of 3–6 screened positive for depression, compared to only 2% (6/329) of participants with an IARSS score of 0–2 (χ^2^ = 62.2, *P* < 0.001).

In unadjusted analyses (**Table 2**), younger age, urban residence, being a Tamil speaker, current alcohol use, and higher IARSS score were associated with an increased risk of probable depression. After multivariable adjustment (**Table 2**), being a Tamil speaker and IARSS score were associated with increased risk of probable depression. The adjusted relative risk ratio for IARSS was 1.83 (95% confidence interval (CI) 1.56–2.14), indicating a nearly two times higher risk of screening positive for depression for each additional point on the IARSS. Turning next to the multivariable linear regression model with continuous depressive symptom severity (PHQ–9 score) as the outcome of interest, we observed a statistically significant positive association between IARSS and depressive symptoms (adjusted *b* = 0.91; 95% CI 0.76–1.06). Put another way, we found an approximate increase in the PHQ–9 score of 0.9 for every additional point on the IARSS.

**Table 2:** Unadjusted and adjusted risk ratios and 95% confidence intervals for variables associated with probable depression

Variable	Unadjusted risk ratio (95% CI)	Adjusted risk ratio (95% CI)
Female (vs other)	0.838 (0.494–1.422)	0.510 (0.248–1.051)
Age, per 10 year	0.725 (0.544–0.964)*	0.802 (0.619–1.037)
Achieved more than primary education (vs other)	1.012 (0.584–1.753)	1.094 (0.631–1.900)
Married (vs unmarried)	0.681 (0.400–1.158)	1.037 (0.604–1.780)
Employed (vs unemployed)	0.917 (0.503–1.672)	0.996 (0.511–1.942)
Urban residence (vs rural)	2.150 (1.270–3.641)*	1.298 (0.772–2.182)
Tamil speaker (vs other)	2.739 (1.623–4.621)**	1.986 (1.196–3.297)*
Current alcohol use	2.156 (1.205–3.859)*	1.807 (0.954–3.422)
IARSS score	1.907 (1.647–2.208)*	1.828 (1.559–2.143)**

CI – confidence interval, IARSS – Internalized AIDS–Related Stigma Scale

*P < 0.05 and ** *P* < 0.001.

In the sensitivity analysis exploring the robustness of the relationship between IARSS and depressive symptoms (PHQ–9 score), we assumed a maximum R–squared value of 0.267 (the R–squared obtained in the multivariable regression model) × 1.3 = 0.347, following the procedures described by Oster [53]. Using this maximum R–squared value, we calculated a delta of 2.34, indicating that confounding by unobserved variables would need to be more than twice as important as confounding by the observed variables in the regression model to generate a regression coefficient for IARSS equal to zero.

## DISCUSSION

In this sample of newly diagnosed PLHIV entering into HIV care in southern India, we found a high prevalence of internalized stigma despite a relatively low prevalence of probable depression. The prevalence of probable depression in this study was 10%, which was lower than prior studies of Indian PLHIV [30,31,54]. The reasons for this lower prevalence of probable depression are unclear. One possible explanation may relate to a temporal trend in rising mean CD4^+^ cell count among patients presenting to YRG CARE, from approximately 160 in 2006 (unpublished data) to 347 in this cohort. In one study of Ugandan PLHIV from 2005–2012, there was a decline over time in mean depression symptom severity scores at ART initiation, a trend that appeared to be explained by improved physical health scores over time [55].

In contrast, we found that internalized stigma was reported commonly, with more than four–fifths of respondents endorsing at least one measure of internalized stigma. While relatively few respondents felt “guilty,” “dirty,” “ashamed,” or “worthless” because of their serostatus, approximately four–fifths of respondents endorsed concerns about serostatus disclosure. This suggests that while most Indian PLHIV may not regard stigmatizing attitudes towards PLHIV as valid, they fear the consequences of disclosure and anticipate rejection and isolation from others [56]. This pattern is consistent with prior studies from India, which have found that PLHIV anticipate stigma much more frequently than they actually experience instances of enacted stigma [28]. Of note, while others have contended that the consequences of disclosure may be particularly harsh for Indian women with HIV, who may face financial hardship and rejection at the hands of husbands’ families [57,58], we did not find significant gender differences in IARSS score or in responses to the items on serostatus disclosure. This contrasts with prior research with Indian PLHIV, which has found higher internalized stigma scores among men, primarily driven by gender differences in feelings of shame or guilt [27,33].

We also found that internalized stigma and depression were closely correlated. Nearly a quarter of participants with an IARSS score of 3–6, but only 2% with an IARSS score of 0–2, screened positive for depression. In a modified Poisson regression model adjusted for sociodemographic variables, we found that each additional point on the IARSS was associated with a nearly two times higher risk of probable depression. Our sensitivity analysis demonstrated that this finding was fairly robust, in that confounding by unobserved variables would need to be more than twice as important as confounding by observed variables to generate a regression coefficient for IARSS equal to zero. A person living with HIV who has internalized and accepted negative attitudes towards PLHIV as valid may suffer from self–hatred [59], hopelessness [60], isolation, and emotional distress [61]. Furthermore, a person living with HIV who harbors concerns about disclosure may lack social support and affirmation from family and friends. Fears of disclosure among Indian PLHIV have been linked to depressive symptoms [28] as well as poor outcomes such as failure to link to HIV care [9].

Further study is needed to determine whether depression and internalized stigma are associated with poor HIV treatment outcomes such as loss to care and lack of virologic suppression in India, and in particular whether the combination of depression and stigma is particularly deleterious. Such studies would highlight the importance of screening PLHIV for internalized stigma and depression at entry into care and ultimately reveal targets for policymakers to forestall loss from the HIV care continuum. For example, if fears of disclosure and the potential consequences of social rejection and economic incapacity are driving depressive symptoms and poor HIV–related outcomes, then interventions to help PLHIV safely disclose their serostatus or to bolster their economic capacity may help to keep PLHIV in care and on treatment.

There are several limitations to this study. First, as the study was conducted at a single HIV care center in India, the findings may not be generalizable to the entire country. In particular, given the high proportion of patients at YRG CARE who self–identify as heterosexual (although this number may be affected by disclosure bias), our findings may not be applicable to sexual and gender minorities. Nevertheless, we hope that our results will spur similar research among sexual and gender minorities and PLHIV who seek care in other parts of India and in the public sector. In addition, the type of private, multi–service program available at YRG CARE can be found in other cities in India as well as other Asian countries. Second, although the PHQ–9 can be used to identify persons with symptoms indicative of probable depression, we did not have access to data on DSM–consistent diagnoses of depressive disorders. However, the PHQ–9 has been shown to have good accuracy in diagnosing major depressive disorder in India [40,41]. Third, the IARSS, like other stigma scales, is limited by multiple factors, including 1) the difficulties inherent in operationalizing HIV–related stigma, a concept for which a consensus definition has been elusive [44,62], 2) the inability to draw conclusions on the “prevalence” of internalized stigma as no validated cut–off exists, and 3) the potential conceptual overlap between IARSS and other mental health measures such as the PHQ–9. While we acknowledge that the IARSS may not assess all aspects of internalized stigma among PLHIV and that we cannot comment on an exact “prevalence” of internalized stigma in our sample, our findings suggest an association between depression and the dimensions of internalized stigma in India captured by the IARSS—in particular, fears of serostatus disclosure and self–hatred. Finally, we encountered limitations in the use of the existing YRG CARE questionnaire for correlates of interest (in particular, the dichotomous nature of the alcohol use variable). Further research is needed to more precisely measure these variables, including measures of problem drinking, to understand possible associations with depression among Indian PLHIV.

In summary, we found that PLHIV entering into HIV care at a large ART center in southern India commonly endorsed fears of serostatus disclosure and that stigma and depression were closely correlated. Although there is likely a bidirectional relationship between stigma and depression among Indian PLHIV, as has been demonstrated in other LMICs [10,11], more study is needed to characterize the linkages between HIV–related stigma and depression in India specifically and the impact of these conditions on HIV–related outcomes. Depression and stigma may provide important targets for policymakers seeking to keep Indian PLHIV alive, in care, and on effective treatment.
